# Diet-Related Behaviors and Diet Quality among School-Aged Adolescents Living in Greece

**DOI:** 10.3390/nu12123804

**Published:** 2020-12-11

**Authors:** Vassiliki Benetou, Afroditi Kanellopoulou, Eleftheria Kanavou, Anastasios Fotiou, Myrto Stavrou, Clive Richardson, Philippos Orfanos, Anna Kokkevi

**Affiliations:** 1 Department of Hygiene, Epidemiology and Medical Statistics, School of Medicine, National and Kapodistrian University of Athens, 115-27 Athens, Greece; afkanellopoulou@gmail.com (A.K.); phorfanos@med.uoa.gr (P.O.); 2 Department of Hygiene and Epidemiology, School of Medicine, University of Ioannina, 451-10 Ioannina, Greece; 3 University Mental Health, Neurosciences, & Precision Medicine Research Institute “Costas Stefanis” (UMHRI), 115-27 Athens, Greece; erevna.HBSC@epipsi.gr (E.K.); afotiou@med.uoa.gr (A.F.); erevna.hbsc@epipsi.gr (M.S.); akokkevi@med.uoa.gr (A.K.); 4 Department of Economic and Regional Development, Panteion University of Social and Political Sciences, 176-71 Athens, Greece; crichard@panteion.gr

**Keywords:** diet quality, adolescents, diet-related behaviors, cross-sectional study, breakfast, fast-foods, family

## Abstract

Prevalence of diet-related behaviors (i.e., breakfast consumption, eating with the family) and their association with a 17-point diet quality score, constructed on the basis of reported frequency (in days/week) of vegetable, fruit, sweets and sugar-sweetened beverages consumption, was investigated among 3525 adolescents (51.5% girls) aged 11, 13 and 15 years, who were participants in the Greek arm of the international Health Behaviour in School-Aged Children (HBSC) cross-sectional study, during 2018. Almost one-third (32.9%) of the sample had breakfast ≤1 day/weekdays, 20.2% rarely ate with the family, 26.1% had a meal while watching TV ≥5 days/week, 31.7% had a snack in front of a screen ≥5 days/week and 24.1% ate in fast-food restaurants at least once/week. Multivariable ordinal logistic regression revealed that eating breakfast ≤1 day/weekdays compared to 4–5 days/weekdays (Odds ratio (OR): 1.56, 95% con-fidence interval (CI): 1.34–1.82), eating rarely with the family compared to almost every day (OR: 1.35, 95% CI: 1.13–1.60) and eating in fast-food restaurants ≥2 times/week vs. rarely (OR: 4.59, 95% CI: 3.14–6.70) were associated with higher odds of having poor diet quality. High frequency of having meals/snacks in front of a screen/TV was also associated with poor diet quality. Efforts to prevent or modify these behaviors during adolescence may contribute to healthier diet.

## 1. Introduction

Nutrition plays a fundamental role in the development of each child’s full potential and influences their later susceptibility to chronic diseases [1,2]. Especially during adolescence, the second fastest growth period after infancy, adequate nutrition in terms of quantity and quality is essential in order to meet the increased demands of pubertal growth and development and achieve optimal physical, cognitive and mental health [3]. Furthermore, as dietary habits and diet-related behaviors are formed and established during this period, adolescence offers a unique opportunity to develop and promote healthy dietary habits and behaviors [4]. Family and peers, school, mass media and advertising, as well as the socio-economic and cultural environment are among the contributing factors in the formation of diet-related behaviors and food choices [5].

Skipping breakfast, snacking in front of a screen, eating in fast-food restaurants and eating infrequently with the family are, in general, highly prevalent behaviors among adolescents and have been linked to both unhealthy diets and adverse health outcomes [6,7,8,9,10,11,12,13,14]. Skipping breakfast has been associated with excess adiposity, worse blood lipid profile and blood pressure levels, insulin-resistance and metabolic syndrome, whereas regular breakfast consumption has been associated with better diet quality and improved cognitive and academic performance among school-aged children and adolescents [6,8,9,10]. Excessive time spent in front of a screen (e.g., smartphones, tablets, computers and TV) has been associated with increased intake of sugar-sweetened beverages, fast-foods, sweets and salty snacks as well as higher total energy intake and higher risk of childhood obesity [7]. Regular fast-food consumption has been associated with regular consumption of sweets/candies and sugar-sweetened beverages, as well as higher total energy intake and poorer diet quality [11,12,13]. On the other hand, having frequent family meals has been associated with consumption of healthy food groups and better diet quality [14,15,16].

Existing data on the prevalence of the above-mentioned diet-related behaviors among children and adolescents in Greece are relatively old, while research has focused more on associations with obesity and relevant biomarkers and less on associations with dietary factors or diet quality [17,18,19,20,21]. Findings based on the most recent study conducted during 2015 among 177,091 students aged 8–17 years living in Greece revealed that skipping breakfast reached 22.4% and 23.1% among boys and girls, respectively, and was associated with poor diet as assessed by the Mediterranean Diet Quality Index for children and adolescents [17]. In the same study, 23.0% of boys and 16.0% of girls reported consuming fast-foods more than once/week, whereas frequent fast-food consumption was strongly correlated with unhealthy dietary habits such as consuming sweets/candy regularly [19]. Furthermore, increased screen time was also associated with lower adherence to the Mediterranean diet [20].

Based on the above, the aim of this study was to provide more recent data on the prevalence of important diet-related behaviors, in particular eating breakfast, eating with the family, snacking or having a meal in front of the TV or a screen and eating in fast-food restaurants, in a representative sample of school-aged adolescents living in Greece and to explore further how these behaviors are linked to consumption of key food groups and beverages for adolescent diet, such as vegetables, fruits, sweets and sugar-sweetened beverages, incorporated in an index that could partly reflect the quality of their diet. We hypothesized that specific diet-related behaviors would be associated with less favorable dietary choices and poorer diet quality, as expressed by the above index, among adolescents.

## 2. Material and Methods

### 2.1. Study Population

Data on eating habits were derived from the 2018 Greek arm of the international Health Behaviour in School-aged Children (HBSC) study [22], a nationally representative survey of 11, 13 and 15-year-old students conducted by the University Mental Health, Neurosciences and Precision Medicine Research Institute quadrennially since 1998. The survey received ethical approval from the Ministry of Education. The study protocol required active parental consent. Prior to survey administration students were informed orally that their participation was voluntary and that they could opt out of filling the survey.

Trained assistants group-administered anonymous questionnaires to 6th, 8th and 10th grade students in the classroom during two consecutive regular class periods. In line with HBSC’s study protocol, a multistage stratified random cluster sampling procedure with the school class as the sampling unit was employed to secure the nationwide sample of students in each year. Stratification was based on (a) administrative region (Nomenclature for territorial units of statistics, 2nd level in the European Union’s classification) (10 out of Greece’s 13 regions, excluding the Ionian and North and South Aegean islands for logistical reasons) and (b) school type (comprehensive/technical/private). The response rate of schools was 93%, while the response rate of students from participating schools was 87%; about 6% of the registered students were absent on the day of administration and 7% of students who were present refused (their parents or themselves) to fill in the questionnaire. Excluding about 8% of the filled-in student questionnaires due to a high proportion of missing values or being outside the age limits imposed by the HBSC survey methodology, the final number of students was 3863 (50.1% girls), of whom 32.0% were 11 years old, 34.0% 13 years old and 34.1% 15 years old, from 238 schools.

### 2.2. Food Consumption and Construction of a Diet Quality Score

A standardized questionnaire with core and optional questions used across the HBSC participating countries included a section for recording food consumption. Specifically, students were asked to assess their frequency of consumption during a typical week for the following food groups and beverages: (a) fruits, (b) vegetables/salads, (c) sweets (including chocolates and candies), (d) sugar-sweetened beverages, (e) coffee, and (f) energy drinks (the last two being national items). The available responses were seven ranging from “never” to “more than once a day, every day”.

We further combined the information on the consumption of four food groups/items: vegetables/salads, fruits, sweets and sugar-sweetened beverages, to construct a diet quality score, which has the simplicity of expressing several dietary exposures through one variable. The rational of the specific diet score was based on the food-based dietary recommendations included in the Greek National Dietary Guidelines for children and adolescents [23]. Thus, for groups/items that are recommended as part of a healthy everyday diet and in particular vegetables/salads and fruits, the scoring took the values of 0–4, with higher values depicting more frequent consumption (in days/week) and better diet quality as follows: (i) ≤1 day/week = 0; (ii) 2–4 days/week = 1; (iii) 5–6 days/week = 2; (iv) 7 days/week = 3 and (v) >once/day = 4. For food groups/items that are not recommended as part of a healthy everyday diet, that is consumption of sweets and sugar-sweetened beverages, the scoring took the values of 0–4, with higher values depicting less frequent consumption and better diet quality as follows: (i) ≤1 day/week = 4; (ii) 2–4 days/week = 3; (iii) 5–6 days/week = 2; (iv) 7 days/week = 1; (v) >once/day = 0. The total score could take values from 0 to 16, where zero corresponds to the worst possible combination of the four food groups/items, and thus the worst diet quality (i.e., once a week or less often consumption of fruits and vegetables/salads and more than once daily sweets and sugar-sweetened beverages) and 16 corresponds to the best possible combination and the best diet quality (i.e., once a week or less often consumption of sweets and sugar-sweetened beverages and more than once daily consumption of fruits and vegetables/salads). Thus, the higher the diet score, the better the quality of the diet.

### 2.3. Diet-Related Behaviors

Students were also asked to report their frequency of adopting the following diet-related behaviors: (a) eating snacks while watching TV (or DVD/video), (b) eating snacks while sitting in front of a screen for homework or games, (c) eating meals while watching TV, (d) eating in fast-food restaurants, (e) eating meals with their family and (f) eating breakfast, defined as more than a glass of milk or fruit juice (asked separately for schooldays and weekends). The available responses with respect to the first three questions (eating snacks/meals while in front of TV/screen) were six ranging from “never” to “every day”. The available responses with respect to eating with the family were five, ranging from “every day” to “never” and with respect to eating in fast-food restaurants were seven, ranging from “never” to “five or more days/week”. For breakfast, the question about the schooldays was used for this analysis with six available responses (from “never” to “all five days”). The reported responses were further grouped into fewer categories.

### 2.4. Other Covariates

Socio-demographic characteristics were recorded. Students were asked to report the year and month they were born and age groups were computed. They further reported their gender and their country of birth, including those of each of their parents. A family affluence scale (FAS) score was computed using a six-item assessment of common material assets or activities; responses were scored and summed to form an HBSC FAS summary score, which was then used to identify groups of young people in the lowest 20% (low affluence), middle 60% (medium affluence) and highest 20% (high affluence) [24].

With respect to physical activity, students were asked to report the number of days over the past week during which they were physically active for a total of at least 60 minutes. Physical activity was defined as any activity that increases the heart rate and makes the person get out of breath some of the time, with examples provided. There were eight response options, ranging from “none” to “7 days”. Height in centimeters (without shoes) and weight in kilograms (without clothes) were self-reported and body mass index (BMI) was calculated as the ratio of weight in kilograms divided by the square of height in meters (kg/m^2^).

### 2.5. Statistical Analysis

Participants’ data were summarized by mean and standard deviation or median and interquartile range for continuous variables, and by frequencies and percentages for categorical variables. For continuous variables, differences between boys and girls (in total and by age group) were assessed via two-sample *t*-test and differences between age groups via one-way ANOVA. For categorical variables, relevant differences were assessed by Pearson’s chi-square test.

The diet quality score was used as a categorical variable divided into three categories: good, moderate and poor. These categories corresponded to the dietary score’s distribution tertiles as follows: poor: 0–9, moderate: 10–12 and good: 13–16.

In order to assess the association of diet quality score with diet-related behaviors ordinal logistic regression was applied. Unadjusted and adjusted odds ratios (OR) (obtained from univariable and multivariable regression analyses, respectively) and their 95% confidence intervals (CI) were computed using as reference category the good diet quality category. This was decided in order to identify behaviors associated with increased odds of not having a good diet quality score, as more easily interpretable and transferable to public health messages. In all models, standard errors were computed allowing for intragroup correlation (Clustered Sandwich Estimator) since non-independence of participants resulting from cluster sampling was suspected. Additional variables included in the final multivariable ordinal logistic regression model were gender, age group, and physical activity in the past 7 days (number of days), as well as place of birth, and family affluence which had been found to be associated (with *p* < 0.20) with diet quality in the univariable analyses. To further evaluate whether the association varied by gender we stratified the final multivariable analysis by gender. In order to assess the possible influence of BMI in our final multivariable model, we also ran an analysis on the sample of 3386 students with available data on BMI. All statistical analyses were conducted using STATA v13.1 (STATA Corporation, College Station, TX, USA).

## 3. Results

From the initial sample of 3863 school-aged adolescents who participated in the 2018 survey, 338 were excluded due to missing values in one or more variables of interest. Thus, the final sample consisted of 3525 adolescents, 1708 boys and 1817 girls, evenly distributed across predefined age groups (Table 1). Half of the participants came from the two prefectures where the largest cities of Greece are situated (37.7% from Attica where Athens/Piraeus is located, 14.7% from Thessaloniki prefecture where Thessaloniki is located). The vast majority (96.6%) were born in Greece, while more than 80% had both parents of Greek origin.

In Table 2, diet-related and lifestyle behaviors are presented in the total sample and by gender in the total sample and among 11-, 13- and 15-years old samples. More than half of the sample (52.5%) had breakfast almost every weekday, but 32.9% had it just one weekday or less often. The frequency of eating breakfast on weekdays differed by gender in the total sample (*p* = 0.001) and among the 15-year old sample (*p* = 0.006), with girls skipping breakfast (defined here as eating breakfast ≤1 day/week) more often than boys (34.6% vs. 31.1% in the total sample, 34.7% vs. 29.1% among the 15-year old sample). Eating with the family almost every day was a frequent habit reaching 79.8% for the total sample, 82.0% for boys and 77.7% for girls. The frequency of eating with the family differed significantly by gender among the total population (*p* = 0.001) and among the 13-year old (*p* = 0.007) and 15-year old students (*p* = 0.001), with fewer girls eating almost every day with their family compared to boys (75.0% vs. 81.4% in the 13-year olds and 72.7% vs. 80.8% in the 15-year olds).

A total of 26.1% of the adolescents ate a meal and a total of 21.7% ate a snack almost every day while watching TV. Almost one-third (31.7%) of the sample reported eating snacks in front of a PC/tablet/laptop 5 or more days per week, with boys reporting this behavior in higher percentages than girls in all age groups. Eating in fast-food restaurants at least twice per week was reported by 6.6% of the total sample, with more boys than girls in all age groups except for the 11-year olds (*p* = 0.448). Physical activity in the past 7 days was also higher among boys compared to girls in the total population and across the age groups.

When differences between the age groups were studied, eating rarely with the family, eating in fast-food restaurants at least once per week and eating meals while watching TV almost every day were all more prevalent among the 15-year-olds compared to the other two younger groups (Supplementary Table S1). Eating frequently snacks in front of a PC/tablet was more prevalent among the 13-year olds whereas eating frequently snacks while watching TV more prevalent among the 11-year olds. Skipping breakfast did not differ across the age-groups. The 15-year olds had lower physical activity levels in comparison to 11- and 13-year olds (Supplementary Table S1).

In Table 3, frequency of consumption of specific food items, components of the diet quality score, as well as the diet quality score in the whole population, and by gender among 11-, 13- and 15-year olds is presented. The majority of the total population did not consume fruits (69.3%) or vegetables (66.3%) every day and boys reported eating fruits and vegetables less often compared to girls (*p* = 0.001). With respect to consumption of sweets and sugar-sweetened beverages, daily consumption reached 15.3% for sweets and 5.7% for the sugar-sweetened beverages, in the total population. More girls than boys reported frequent consumption of sweets, although only in the 15-year old group (*p* = 0.025), whereas boys consumed more sugar-sweetened beverages than girls in all age-groups. Overall, 17.9% of the population was classified in the good diet quality category, 39.4% in the moderate and 42.7% in the poor diet quality category. Girls had a statistically significantly better diet quality than boys in all age groups. When differences between the age groups were studied, consumption of fruits and vegetables was lower and consumption of sweets and sugar-sweetened beverages was higher in the 13- and 15-year old age groups compared to the 11-year olds. Consequently, the 15-year-old adolescents had worse diet quality compared to the younger age groups (*p* < 0.001) (Supplementary Table S2).

In Table 4 odds ratios and 95% confidence intervals, derived from the multivariable ordinal logistic regression, assessing the association of diet-related behaviors and other characteristics with diet quality score (using the good diet quality as a reference), are presented.

Boys had 37% higher odds of having moderate or poor diet quality compared to girls (OR:1.37, 95% CI: 1.20–1.56) and the 15-year olds had 53% higher odds of having moderate or poor diet quality compared to the 11-year olds (OR:1.53, 95% CI: 1.27–1.83). Having breakfast once or less often during the weekdays compared to almost every weekday was associated with 56% higher odds of having worse diet quality (≤1 day/weekdays vs. 4–5 days, OR:1.56, 95% CI: 1.34–1.82). Similarly, eating rarely with the family compared to almost every day, was associated with higher odds of having worse diet quality (OR: 1.35, 95% CI: 1.13–1.60). The frequency of eating snacks or meals while watching TV was positively associated with poorer diet quality, as well as eating snacks in front of a PC, tablet or laptop. Eating in fast-food restaurants 1–3 days per month was associated with higher odds of having a moderate or poor diet quality compared to eating rarely. Notably, adolescents eating in fast-food restaurants at least twice per week had almost 4.6 times higher odds of exhibiting a moderate or poor diet quality compared to those eating rarely in fast food restaurants (OR: 4.59, 95% CI: 3.14–6.70). When data were stratified by gender, eating rarely with the family and eating snacks while watching TV were associated with worse diet quality only among girls (Supplementary Table S3). All other associations remained significant in boys and girls, whereas eating in fast-food restaurants was associated with much higher odds of having a worse diet quality (OR: 8.12, 95% CI: 4.34–15.18) in girls compared to boys (OR: 3.41, 95% CI: 2.15–5.41). The results from the multivariable analysis for the association between diet-related behaviors and other characteristics and diet quality score after including BMI in the model were practically identical (Supplementary Table S4).

## 4. Discussion

In this large sample of school-aged adolescents living in Greece, relatively high proportions, ranging from 20.2% to 32.9%, were regularly skipping breakfast, were frequently eating in front of a screen and in fast-food restaurants, and rarely eating with the family. Differences between boys and girls and across the age-groups were evident in some of the studied diet-related behaviors. Skipping breakfast, eating frequently in front of a screen and in fast-food restaurants, and eating rarely with the family were all independently associated with worse diet quality, assessed by a diet score incorporating frequency of fruit, vegetables, sweets and sugar-sweetened beverages consumption. Associations remain unchanged after controlling for important possible confounders such as physical activity, family affluence and body mass index.

Almost one-third of this sample, more girls than boys, had breakfast only once, or less often, during the week. Skipping breakfast is a frequent finding in similar studies conducted in Greece and around the world [8,17,18]. In a systematic review collecting data from 286,804 children and adolescents living in 33 countries during 2008–2018, most studies reported that at least 10–30% of children and adolescents never eat breakfast, with an increasing trend in adolescents and mostly in girls [8]. The main reasons that have been proposed are the lack of time and not feeling hungry in the morning, as well as the effort to control body weight, the latter especially among girls. It should be noted that skipping breakfast has been defined differently across the studies, an issue that adds complexity to the comparisons among studies and the interpretation of the results. In this study, adolescents skipping regular breakfast consumption (defined as eating something more than one glass of milk or juice in the morning), had worse diet quality in comparison to those eating regularly breakfast during the week. There is consistent evidence that breakfast forms an integral part of a healthy diet that positively impacts health and well-being in children [6,8,9,10,25,26] and in adults [27]. In a cross-sectional study from Belgium with 341 adolescents 13–18 years old, consumers of a good-quality breakfast had a better overall dietary pattern, both on a nutrient and food group level, in comparison to consumers of a low-quality breakfast [25]. In a representative sample of 4200 children, 2–18 years old, from the National Health and Nutrition Examination Survey in US during 2001–2008, consumers of most breakfast patterns had higher intakes of key nutrients (dietary fiber, vitamin D, calcium and potassium), as well as better diet quality indexes compared to breakfast skippers [26]. The importance of consuming a healthy breakfast every day is stressed in the Greek National Dietary Guidelines for infants, children and adolescents established in 2014 [23]. According to these guidelines, breakfast comprises one of the most important meals of the day and children are recommended to consume foods from the following three food groups at minimum: dairy products, cereals (preferably whole grain) and fruits or vegetables.

In this study, a relatively high proportion of adolescents (26.1%) had a meal while watching TV and 31.7%, more boys than girls, had a snack in front of a screen, five or more days per week. Furthermore, frequency of eating snacks in front of a screen (PC, tablet or laptop) or meals while watching TV was positively associated with poorer diet quality. In the context of the Greek arm of the HBSC study conducted 20 years ago (1997–1998), in a sample of 4211 students, TV viewing was positively associated with consumption of sodas, crisps, cakes, pastries, sweets and chocolates at least once a day, but negatively associated with consumption of fruits [21]. Other studies have also reported an association between high screen time and less healthy dietary patterns, such as less fruit and vegetable consumption and greater consumption of energy-dense snacks and sugar-sweetened beverages in all age groups [28,29,30].

Frequency of eating in fast-food restaurants at least once a week among this study sample was high (24.1%), with more boys than girls and more 15-year olds reporting this behavior. Furthermore, eating frequently in fast-food restaurants was the behavior with the highest odds of having poor diet quality, especially among girls, compared to other studied behaviors. Previous studies investigating fast-food consumption in relation to diet quality among adolescents in Greece, and elsewhere, have reported similar associations [11,12,13,19]. In most studies, fast-food consumption has been associated with higher availability and intake of sugar-sweetened beverages and French fries and lower intake of milk, fruit and vegetables, as well as a lower likelihood of being served vegetables or milk with home meals [13]. Consumption of fast-foods may also be a surrogate marker for other unhealthy behaviors associated with poor diet quality and dietary choices [31].

A high proportion of participants in this sample, reaching 80%, had a family meal almost every day, while 20% rarely had a family meal. Eating rarely with the family was associated with worse diet quality, a consistent finding across similar studies [14,15,16]. The healthier food choices associated with eating with the family are possibly due to the consumption of higher quality of foods, as well as increased opportunity to have family discussions about healthy eating and dietary choices [32]. A large proportion of the adolescents in this sample, more than 60%, did not adhere to the daily consumption of fruits and vegetables recommended by the Greek food-based dietary guidelines for children and adolescents. According to these guidelines, consumption of a variety of fruits and vegetables several times per day in every main meal is recommended [23]. Frequent fruit and vegetable consumption is essential during childhood and beyond, contributing to optimal diet and nutritional status, lowering risk of chronic diseases and promoting long-term health [33]. On the other hand, the proportion of adolescents who reported daily consumption of sweets and sugar-sweetened beverages was not negligible. Consumption of added sugars, including sweetened beverages and soft drinks, is associated with an increased risk of dental caries and childhood overweight/obesity. According to the national guidelines for children and adolescents, sweets should be consumed in moderation and during special occasions, whereas sugar-sweetened beverages should be avoided [23]. Based on the diet quality index and its three categories constructed by us, the majority of adolescents (82.0%) were classified in the moderate and poor diet quality categories with girls scoring better than boys in the younger age groups. Older adolescents had a worse diet quality compared to their younger counterparts. In the previous analysis of the Greek segment of the HBSC study in 1997–1998, similar results were found with respect to diet quality [21]. Girls had better diet quality compared to boys and older age was positively correlated with poor diet quality. Nevertheless, the diet quality index used in that study was defined differently, with more food groups available to combine in the index.

Our study has several limitations. First, the cross-sectional design of the study did not allow inference of causal associations between diet-related behaviors and diet quality. Second, information about diet was assessed through a non-quantitative food frequency questionnaire, which formed part of the HSBC questionnaire, with a limited number of food items, not allowing a comprehensive study of diet, both in terms of quantity as well as in terms of quality. In that respect, we could not use any of the existing validated diet quality scores for children and adolescents, and instead, we have tried to construct a diet quality score to capture adherence to a healthy dietary pattern combining in one variable, the frequent consumption of fruit and vegetables and the less frequent consumption of sweets and sugar-sweetened beverages. Nevertheless, this diet quality score was neither an established, nor a validated diet quality index and it was based on a limited number of items. All information collected was self-reported by adolescents, introducing a degree of information bias into the study. Residual confounding is also possible since variables that have not been collected or not adequately measured, were not accounted for in the analysis. On the other hand, advantages of our study are the large sample size, the standardized methodology in the context of an established multinational survey allowing comparisons to other participating countries, and the random sampling allowing generalizing our findings to the adolescent population in Greece. Furthermore, data were collected during 2018, making findings deriving from this analysis quite current.

In conclusion, we found evidence that specific diet-related behaviors such as skipping breakfast, eating meals/snacks in front of a screen, eating in fast-food restaurants and eating infrequently with the family were quite prevalent in a representative sample of school-aged adolescents living in Greece and that all these behaviors were associated with less favorable diet quality. Modifying or preventing the establishment of these behaviors during childhood and adolescence through the implementation of targeted, multifaceted health promotion and education initiatives may lead to healthier dietary choices and better diet quality.

## Figures and Tables

**Table 1:** Socio-demographic and baseline characteristics of 3525 participants in the 2018 Greek arm of the Health Behaviour of School-Aged Children Study.

Characteristics	*n* (%)
Gender Boys	1708 (48.5)
Girls	1817 (51.5)
Age group	
11-year olds	1085 (30.8)
13-year olds	1220 (34.6)
15-year olds	1220 (34.6)
Grade	
6th	1106 (31.4)
8th	1228 (34.8)
10th	1191 (33.8)
Region/municipality	
Attica	1328 (37.7)
Thessaloniki	519 (14.7)
Other	1678 (47.6)
Place of birth	
Greece	3404 (96.6)
Other	121 (3.4)
Mother’s place of birth ^a^	
Greece	2817 (80.2)
Other	694 (19.8)
Father’s place of birth ^a^	
Greece	2940 (83.8)
Other	568 (16.2)
Body mass index ^b^, kg/m^2^, mean (sd)	20.3 (3.5)
Physical activity in the past 7 days, days, mean (sd)	4.0 (2.0)
Family affluence scale (FAS) score ^c^	
Low 20% affluent	504 (14.3)
Middle 60% affluent	2258 (64.1)
High 20% affluent	763 (21.6)

Abbreviation: standard deviation (sd). ^a^ Number of students does not add up to 3525, due to missing values. ^b^ Available data for 3386 students. ^c^ Quantiles were calculated based on FAS score distribution of FAS score by gender and age group.

**Table 2:** Diet-related and lifestyle behaviors and characteristics of 3525 participants in the 2018 Greek arm of the Health Behaviour of School-Aged Children Study for the total sample and by age group and gender.

Behaviors Characteristics	Total	All Ages (*n* = 3525)			11-Year Olds (*n* = 1085)			13-Year Olds (*n* = 1220)			15-Year Olds (*n* = 1220)		
		Boys (*n* = 1708)	Girls (*n* = 1817)	*p*-Value ^+^	Boys (*n* = 523)	Girls (*n* = 562)	*p*-Value ^+^	Boys (*n* = 591)	Girls (*n* = 629)	*p*-Value ^+^	Boys (*n* = 594)	Girls (*n* = 626)	*p*-Value ^+^
Eating breakfast on weekdays, days/week, *n* (%)				0.001			0.306			0.197			0.006
≤1	1160 (32.9)	531 (31.1)	629 (34.6)		167 (31.9)	187 (33.3)		191 (32.3)	225 (35.8)		173 (29.1)	217 (34.7)	
2–3	516 (14.6)	227 (13.3)	289 (15.9)		59 (11.3)	78 (13.9)		82 (13.9)	98 (15.6)		86 (14.5)	113 (18.1)	
4–5	1849 (52.5)	950 (55.6)	899 (49.5)		297 (56.8)	297 (52.8)		318 (53.8)	306 (48.6)		335 (56.4)	296 (47.3)	
Eating with family, *n* (%)				0.001			0.357			0.007			0.001
Rarely	713 (20.2)	307 (18.0)	406 (22.3)		83 (15.9)	78 (13.9)		110 (18.6)	157 (25.0)		114 (19.2)	171 (27.3)	
Almost everyday	2812 (79.8)	1401 (82.0)	1411 (77.7)		440 (84.1)	484 (86.1)		481 (81.4)	472 (75.0)		480 (80.8)	455 (72.7)	
Eating snacks while watching TV, days/week, *n* (%)				0.050			0.604			0.056			0.361
<1	1214 (34.4)	580 (34.0)	634 (34.9)		158 (30.2)	183 (32.6)		208 (35.2)	215 (34.2)		214 (36.0)	236 (37.7)	
1–2	948 (26.9)	434 (25.4)	514 (28.3)		140 (26.8)	144 (25.6)		136 (23.0)	185 (29.4)		158 (26.6)	185 (29.6)	
3–4	598 (17.0)	316 (18.5)	282 (15.5)		104 (19.9)	97 (17.3)		108 (18.3)	93 (14.8)		104 (17.5)	92 (14.7)	
≥5	765 (21.7)	378 (22.1)	387 (21.3)		121 (23.1)	138 (24.6)		139 (23.5)	136 (21.6)		118 (19.9)	113 (18.1)	
Eating snacks in front of PC/tablet/laptop, days/week, *n* (%)				<0.001			<0.001			<0.001			<0.001
<1	1219 (34.6)	479 (28)	740 (40.7)		152 (29.1)	239 (42.5)		162 (27.4)	246 (39.1)		165 (27.8)	255 (40.7)	
1–2	661 (18.8)	292 (17.1)	369 (20.3)		105 (20.1)	126 (22.4)		94 (15.9)	114 (18.1)		93 (15.7)	129 (20.6)	
3–4	527 (15.0)	292 (17.1)	235 (12.9)		96 (18.4)	76 (13.5)		93 (15.7)	70 (11.1)		103 (17.3)	89 (14.2)	
≥5	1118 (31.7)	645 (37.8)	473 (26.0)		170 (32.5)	121 (21.5)		242 (40.9)	199 (31.6)		233 (39.2)	153 (24.4)	
Eating meals while watching TV, days/week, *n* (%)				0.155			0.915			0.271			0.601
<1	1288 (36.5)	594 (34.8)	694 (38.2)		185 (35.4)	208 (37.0)		197 (33.3)	239 (38.0)		212 (35.7)	247 (39.5)	
1–2	754 (21.4)	383 (22.4)	371 (20.4)		133 (25.4)	135 (24.0)		139 (23.5)	126 (20.0)		111 (18.7)	110 (17.6)	
3–4	563 (16.0)	272 (15.9)	291 (16.0)		84 (16.1)	93 (16.5)		94 (15.9)	103 (16.4)		94 (15.8)	95 (15.2)	
≥5	920 (26.1)	459 (26.9)	461 (25.4)		121 (23.1)	126 (22.4)		161 (27.2)	161 (25.6)		177 (29.8)	174 (27.8)	
Eating in fast-food restaurants, *n* (%)				<0.001			0.448			0.049			<0.001
Rarely	1171 (33.2)	547 (32.0)	624 (34.3)		225 (43.0)	250 (44.5)		175 (29.6)	199 (31.6)		147 (24.7)	175 (28)	
1–3 days per month	1505 (42.7)	693 (40.6)	812 (44.7)		216 (41.3)	242 (43.1)		261 (44.2)	284 (45.2)		216 (36.4)	286 (45.7)	
Once per week	617 (17.5)	320 (18.7)	297 (16.3)		60 (11.5)	54 (9.6)		107 (18.1)	119 (18.9)		153 (25.8)	124 (19.8)	
At least twice per week	232 (6.6)	148 (8.7)	84 (4.6)		22 (4.2)	16 (2.8)		48 (8.1)	27 (4.3)		78 (13.1)	41 (6.5)	
Family affluence scale (FAS) score ^a^, *n* (%)				0.228			0.737			0.398			0.211
Least (20%) affluent	504 (14.3)	245 (14.3)	259 (14.3)		71 (13.6)	83 (14.8)		95 (16.1)	87 (13.8)		79 (13.3)	89 (14.2)	
Middle (60%) affluent	2258 (64.1)	1073 (62.8)	1185 (65.2)		325 (62.1)	352 (62.6)		357 (60.4)	402 (63.9)		391 (65.8)	431 (68.8)	
Highest (20%) affluent	763 (21.6)	390 (22.8)	373 (20.5)		127 (24.3)	127 (22.6)		139 (23.5)	140 (22.3)		124 (20.9)	106 (16.9)	
Physical activity in the past 7 days, days, mean (sd)	4.0 (2.0)	4.3 (2.0)	3.7 (2.0)	<0.001	4.6 (2.0)	4.3 (1.8)	0.005	4.4 (2.0)	3.6 (2.0)	<0.001	4.0 (2.1)	3.4 (2.0)	<0.001

Abbreviation: standard deviation (sd). ^a^ Quantiles were calculated based on FAS score distribution of FAS score by gender and age group. ^+^ *p*-values from Pearson’s X^2^-test for categorical variables and two-sample *t*-test for continuous variables.

**Table 3:** Frequency of consumption of specific food items, mean diet quality score and categories of diet quality score of 3525 participants in the 2018 Greek arm of the Health Behaviour of School-Aged Children Study for the total sample and by age group and gender.

Food Items/Diet Quality Score	Total	All Ages (*n* = 3525)			11-Year Olds (*n* = 1085)			13-Year Olds (*n* = 1220)			15-Year Olds (*n* = 1220)		
		Boys (*n* = 1708)	Girls (*n* = 1817)	*p*-Value ^+^	Boys (*n* = 523)	Girls (*n* = 562)	*p*-Value ^+^	Boys (*n* = 591)	Girls (*n* = 629)	*p*-Value ^+^	Boys (*n* = 594)	Girls (*n* = 626)	*p*-Value ^+^
Fruits intake, days/week, *n* (%)				0.001			0.779			0.006			0.023
≤1	690 (19.6)	317 (18.6)	373 (20.5)		73 (14.0)	83 (14.8)		110 (18.6)	134 (21.3)		134 (22.6)	156 (24.9)	
2–4	1186 (33.6)	595 (34.8)	591 (32.5)		161 (30.8)	157 (27.9)		211 (35.7)	211 (33.5)		223 (37.5)	223 (35.6)	
5–6	566 (16.1)	307 (18.0)	259 (14.3)		90 (17.2)	98 (17.4)		111 (18.8)	76 (12.1)		106 (17.8)	85 (13.6)	
7	679 (19.3)	294 (17.2)	385 (21.2)		114 (21.8)	137 (24.4)		112 (19.0)	143 (22.7)		68 (11.4)	105 (16.8)	
>once daily	404 (11.5)	195 (11.4)	209 (11.5)		85 (16.3)	87 (15.5)		47 (8.0)	65 (10.3)		63 (10.6)	57 (9.1)	
Vegetables intake, days/week, *n* (%)				<0.001			0.049			0.001			0.226
≤1	679 (19.3)	377 (22.1)	302 (16.6)		129 (24.7)	99 (17.6)		124 (21.0)	96 (15.3)		124 (20.9)	107 (17.1)	
2–4	913 (25.9)	459 (26.9)	454 (25.0)		119 (22.8)	128 (22.8)		163 (27.6)	155 (24.6)		177 (29.8)	171 (27.3)	
5–6	743 (21.1)	358 (21.0)	385 (21.2)		95 (18.2)	106 (18.9)		135 (22.8)	126 (20.0)		128 (21.5)	153 (24.4)	
7	813 (23.1)	353 (20.7)	460 (25.3)		122 (23.3)	160 (28.5)		119 (20.1)	174 (27.7)		112 (18.9)	126 (20.1)	
>once daily	377 (10.7)	161 (9.4)	216 (11.9)		58 (11.1)	69 (12.3)		50 (8.5)	78 (12.4)		53 (8.9)	69 (11.0)	
Sweets intake, days/week, *n* (%)				0.086			0.428			0.155			0.025
≤1	1350 (38.3)	667 (39.1)	683 (37.6)		249 (47.6)	274 (48.8)		219 (37.1)	220 (35.0)		199 (33.5)	189 (30.2)	
2–4	1178 (33.4)	564 (33.0)	614 (33.8)		167 (31.9)	164 (29.2)		191 (32.3)	210 (33.4)		206 (34.7)	240 (38.3)	
5–6	455 (12.9)	234 (13.7)	221 (12.2)		41 (7.8)	61 (10.9)		93 (15.7)	79 (12.6)		100 (16.8)	81 (12.9)	
7	319 (9.0)	133 (7.8)	186 (10.2)		41 (7.8)	40 (7.1)		47 (8.0)	72 (11.4)		45 (7.6)	74 (11.8)	
>once daily	223 (6.3)	110 (6.4)	113 (6.2)		25 (4.8)	23 (4.1)		41 (6.9)	48 (7.6)		44 (7.4)	42 (6.7)	
Sugar-sweetened beverage intake, days/week, *n* (%)				<0.001			0.009			<0.001			<0.001
≤1	2477 (70.3)	1078 (63.1)	1399 (77.0)		394 (75.3)	470 (83.6)		355 (60.1)	455 (72.3)		329 (55.4)	474 (75.7)	
2–4	679 (19.3)	416 (24.4)	263 (14.5)		81 (15.5)	55 (9.8)		160 (27.1)	106 (16.9)		175 (29.5)	102 (16.3)	
5–6	168 (4.8)	104 (6.1)	64 (3.5)		25 (4.8)	14 (2.5)		30 (5.1)	30 (4.8)		49 (8.2)	20 (3.2)	
7	111 (3.1)	60 (3.5)	51 (2.8)		13 (2.5)	12 (2.1)		28 (4.7)	23 (3.7)		19 (3.2)	16 (2.6)	
>once daily	90 (2.6)	50 (2.9)	40 (2.2)		10 (1.9)	11 (2.0)		18 (3.0)	15 (2.4)		22 (3.7)	14 (2.2)	
Diet quality score ^a^, mean (sd)	9.9 (2.9)	9.7 (2.8)	10.1 (2.9)	<0.001	10.4 (2.7)	10.7 (2.7)	0.026	9.5 (2.8)	9.9 (2.8)	0.007	9.2 (2.9)	9.6 (3.0)	0.016
Diet quality groups ^b^, *n* (%)				<0.001			0.014			0.007			0.018
Poor	1504 (42.7)	773 (45.3)	731 (40.2)		184 (35.2)	176 (31.3)		262 (44.3)	261 (41.5)		327 (55.1)	294 (47.0)	
Moderate	1389 (39.4)	676 (39.6)	713 (39.2)		230 (44.0)	226 (40.2)		260 (44.0)	254 (40.4)		186 (31.3)	233 (37.2)	
Good	632 (17.9)	259 (15.2)	373 (20.5)		109 (20.8)	160 (28.5)		69 (11.7)	114 (18.1)		81 (13.6)	99 (15.8)	

Abbreviation: standard deviation (sd). ^a^ Values range from 0 to 16. Zero corresponds to the worst possible diet quality score (once a week or less often fruits and vegetables/salads and more than once daily sweets and sugar-sweetened beverages) and 16 to the best possible diet quality score (once a week or less often sweets and sugar-sweetened beverages and more than once daily fruits and vegetables/salads). ^b^ Calculated using dietary score’s tertiles. Poor includes dietary scores from 0 to 9, moderate includes scores from 10 to 12 and good includes scores from 13 to 16. ^+^ *p*-values from Pearson’s X^2^-test for categorical variables and two-sample *t*-test for continuous variables.

**Table 4:** Adjusted odds ratios (OR) and associated 95% confidence intervals (CI) from multivariable ordinal logistic regression model for the association between diet-related behaviors and other characteristics and diet quality score ^†^ among 3525 participants in the 2018 Greek arm of the Health Behaviour of School-Aged Children Study.

Behaviors/Characteristics	Diet Quality Score ^†^	
	OR (95% CI)	*p*-Value
Gender		
Boys	1.37 (1.20–1.56)	<0.001
Girls	Ref.	
Age group		
11-year olds	Ref.	
13-year olds	1.31 (1.10–1.55)	0.002
15-year olds	1.53 (1.27–1.83)	<0.001
Place of birth		
Greece	Ref.	
Other	1.32 (0.90–1.93)	0.159
Physical activity in the past 7 days, days	0.80 (0.77–0.83)	<0.001
Eating breakfast on weekdays, days/week		
≤1	1.56 (1.34–1.82)	<0.001
2–3	1.17 (0.97–1.42)	0.108
4–5	Ref.	
Eating with family		
Rarely	1.35 (1.13–1.60)	0.001
Almost everyday	Ref.	
Eating snacks while watching TV, days/week		
<1	Ref.	
1–2	1.24 (1.03–1.50)	0.022
3–4	1.45 (1.18–1.78)	<0.001
≥5	1.42 (1.14–1.75)	0.001
Eating snacks in front of PC/laptop/tablet, days/week		
<1	Ref.	
1–2	1.15 (0.96–1.38)	0.136
3–4	1.48 (1.19–1.83)	<0.001
≥5	1.85 (1.55–2.21)	<0.001
Eating meals while watching TV, days/week		
<1	Ref.	
1–2	1.14 (0.96–1.35)	0.136
3–4	1.47 (1.19–1.80)	<0.001
≥5	1.45 (1.21–1.75)	<0.001
Eating in fast-food restaurants		
Rarely	Ref.	
1–3 days per month	1.52 (1.30–1.78)	<0.001
Once per week	2.16 (1.74–2.70)	<0.001
At least twice per week	4.59 (3.14–6.70)	<0.001
Family affluence scale (FAS) score ^a^		
Least affluent 20%	Ref.	
Middle affluent 60%	0.84 (0.70–1.00)	0.056
Highest affluent 20%	0.65 (0.53–0.80)	<0.001

Abbreviation: Reference (Ref.); Odd Ratio (OR); Confidence Interval (CI). ^a^ Quantiles calculated based on FAS score distribution of FAS score by gender and age group. ^†^ Diet quality score (ranging from 0 to 16) is the outcome variable which is divided into three categories: good, moderate and poor. These categories are dietary score’s tertiles calculated as follows; poor: dietary score from 0 to 9, moderate: dietary scores from 10 to 12, good: dietary scores from 13 to 16. Good diet quality category is the reference category in order to identify behaviors associated with increased odds of not having a good diet quality score.

## Supplementary Materials

The following are available online at https://www.mdpi.com/2072-6643/12/12/3804/s1, Table S1. Diet-related and lifestyle behaviors and characteristics by age group in the 2018 Greek arm of the Health Behaviour of School-Aged Children Study; Table S2. Frequency of consumption of specific food items, mean diet quality score and categories of diet quality score by age group in the 2018 Greek arm of the Health Behaviour of School-Aged Children Study; Table S3. Adjusted odds ratios (OR) and associated 95% confidence intervals (CI) from multivariable ordinal logistic regression model for the association between diet-related behaviors and other characteristics and diet quality score by gender in the 2018 Greek arm of the Health Behaviour of School-Aged Children Study; Table S4. Adjusted odds ratios (OR) and associated 95% confidence intervals (CI) from multivariable ordinal logistic regression model further adjusted for body mass index for the association between diet-related behaviors and other characteristics and diet quality score among 3386 participants in the 2018 Greek arm of the Health Behaviour of School-Aged Children Study.
