# Supplementary material for: Diet-Related Behaviors and Diet Quality among School-Aged Adolescents Living in Greece
Source: Nutrients. 2020 Dec 11;12(12):3804. doi: 10.3390/nu12123804 (PMC7763124; doi:10.3390/nu12123804)
Supplement: Supplementary file 1 [file nutrients-12-03804-s001.pdf]

Table S1. Diet-related and lifestyle behaviors and characteristics by age group in the 2018 Greek arm of the Health Behaviour of School-Aged Children Study.

Behaviors/Characteristics	**11-Year Olds (*n* = 1085)**	**13-Year Olds (*n* = 1220)**	**15-Year Olds (*n* = 1220)**	***p*-Value[+]**
Eating breakfast on weekdays, days/week, *n* (%)				0.098
≤1	354 (32.6)	416 (34.1)	390 (32.0)	
2–3	137 (12.6)	180 (14.8)	199 (16.3)	
4–5	594 (54.7)	624 (51.1)	631 (51.7)	
Eating with family, *n* (%)				<0.001
Rarely	161 (14.8)	267 (21.9)	285 (23.4)	
Almost everyday	924 (85.2)	953 (78.1)	935 (76.6)	
Eating snacks while watching TV, days/week, *n* (%)				0.017
<1	341 (31.4)	423 (34.7)	450 (36.9)	
1–2	284 (26.2)	321 (26.3)	343 (28.1)	
3–4	201 (18.5)	201 (16.5)	196 (16.1)	
≥5	259 (23.9)	275 (22.5)	231 (18.9)	
Eating snacks in front of PC/tablet/laptop, days/week, *n* (%)				<0.001
<1	391 (36.0)	408 (33.4)	420 (34.4)	
1–2	231 (21.3)	208 (17.0)	222 (18.2)	
3–4	172 (15.9)	163 (13.4)	192 (15.7)	
≥5	291 (26.8)	441 (36.1)	386 (31.6)	
Eating meals while watching TV, days/week, *n* (%)				0.002
<1	393 (36.2)	436 (35.7)	459 (37.6)	
1–2	268 (24.7)	265 (21.7)	221 (18.1)	
3–4	177 (16.3)	197 (16.1)	189 (15.5)	
≥5	247 (22.8)	322 (26.4)	351 (28.8)	
Eating in fast-food restaurants, *n* (%)				<0.001
Rarely	475 (43.8)	374 (30.7)	322 (26.4)	
1–3 days per month	458 (42.2)	545 (44.7)	502 (41.1)	
Once per week	114 (10.5)	226 (18.5)	277 (22.7)	
At least twice per week	38 (3.5)	75 (6.1)	119 (9.8)	
Family affluence scale (FAS) score[a], *n* (%)				0.032
Least affluent 20%	154 (14.2)	182 (14.9)	168 (13.8)	
Middle affluent 60%	677 (62.4)	759 (62.2)	822 (67.4)	
Highest affluent 20%	254 (23.4)	279 (22.9)	230 (18.9)	
Physical activity in the past 7 days, days, mean (sd)	4.4 (1.9)	4.0 (2.0)	3.7 (2.1)	<0.001

Abbreviation: sd; standard deviation. [a] Quantiles calculated based on FAS score distribution of FAS score by gender and age group. [+] *p*-values from Pearson's X2-test for categorical variables and one-way ANOVA for continuous variables.

Table S2. Frequency of consumption of specific food items, mean diet quality score and categories of diet quality score by age group in the 2018 Greek arm of the Health Behaviour of School-Aged Children Study.

**Food Items/Diet Quality Score**	**11-Year Olds (*n* = 1085)**	**13-Year Olds (*n* = 1220)**	**15-Year Olds (*n* = 1220)**	***p*-Value[+]**
Fruits intake, days/week, *n* (%)				<0.001
≤1	156 (14.4)	244 (20.0)	290 (23.8)	
2–4	318 (29.3)	422 (34.6)	446 (36.6)	
5–6	188 (17.3)	187 (15.3)	191 (15.7)	
7	251 (23.1)	255 (20.9)	173 (14.2)	
>once daily	172 (15.9)	112 (9.2)	120 (9.8)	
Vegetables intake, days/week, *n* (%)				<0.001
≤1	228 (21.0)	220 (18.0)	231 (18.9)	
2–4	247 (22.8)	318 (26.1)	348 (28.5)	
5–6	201 (18.5)	261 (21.4)	281 (23.0)	
7	282 (26.0)	293 (24.0)	238 (19.5)	
>once daily	127 (11.7)	128 (10.5)	122 (10.0)	
Sweets intake, days/week, *n* (%)				<0.001
≤1	523 (48.2)	439 (36.0)	388 (31.8)	
2–4	331 (30.5)	401 (32.9)	446 (36.6)	
5–6	102 (9.4)	172 (14.1)	181 (14.8)	
7	81 (7.5)	119 (9.8)	119 (9.8)	
>once daily	48 (4.4)	89 (7.3)	86 (7.0)	
Sugar-sweetened beverages intake, days/week, *n* (%)				<0.001
≤1	864 (79.6)	810 (66.4)	803 (65.8)	
2–4	136 (12.5)	266 (21.8)	277 (22.7)	
5–6	39 (3.6)	60 (4.9)	69 (5.7)	
7	25 (2.3)	51 (4.2)	35 (2.9)	
>once daily	21 (1.9)	33 (2.7)	36 (3.0)	
Diet quality score[a], mean (sd)	10.6 (2.7)	9.7 (2.8)	9.4 (2.9)	<0.001
Diet quality groups[b]				<0.001
Poor	360 (33.2)	523 (42.9)	621 (50.9)	
Moderate	456 (42.0)	514 (42.1)	419 (34.3)	
Good	269 (24.8)	183 (15.0)	180 (14.8)	

Abbreviation: sd; standard deviation. [a] Values range from 0 to 16. Zero corresponds to the worst possible diet (once a week or less often fruits and vegetables/salads and more than once daily sweets and sugar-sweetened beverages) and 16 to the best possible diet (once a week or less often sweets and sugar-sweetened beverages and more than once daily fruits and vegetables/salads). [b] Calculated using dietary score's tertiles. Poor includes dietary scores from 0 to 9, moderate includes scores from 10 to 12 and good includes scores from 13 to 16. [+] p-values from Pearson's X^2-test for categorical variables and one-way ANOVA for continuous variables.

Table S3. Adjusted odds ratios (OR) and associated 95% confidence intervals (CI) from multivariable ordinal logistic regression model for the association between diet-related behaviors and other characteristics and diet quality score† by gender in the 2018 Greek arm of the Health Behaviour of School-Aged Children Study.

Behaviors/Characteristics	Diet Quality Score†			
	Boys (n = 1708)		Girls (n = 1817)	
	OR (95% CI)	p-Value	OR (95% CI)	p-Value
Age group				
11-year olds	Ref.		Ref.	
13-year olds	1.38 (1.07–1.78)	0.014	1.24 (0.99–1.55)	0.066
15–year olds	1.63 (1.25–2.11)	<0.001	1.43 (1.12–1.82)	0.004
Place of birth				
Greece	Ref.		Ref.	
Other	1.00 (0.59–1.70)	0.990	1.68 (0.97–2.91)	0.067
Physical activity in the past 7 days, days	0.79 (0.76–0.84)	<0.001	0.80 (0.76–0.84)	<0.001
Eating breakfast on weekdays, days/week				
≤1	1.26 (1.02–1.57)	0.035	1.89 (1.52–2.33)	<0.001
2–3	0.96 (0.72–1.27)	0.751	1.40 (1.09–1.79)	0.007
4–5	Ref.		Ref.	
Eating with family				
Rarely	1.22 (0.97–1.53)	0.093	1.46 (1.14–1.87)	0.003
Almost everyday	Ref.		Ref.	
Eating snacks while watching TV, days/week				
<1	Ref.		Ref.	
1–2	1.09 (0.84–1.41)	0.517	1.39 (1.09–1.78)	0.008
3–4	1.29 (0.97–1.71)	0.080	1.62 (1.20–2.20)	0.002
≥5	1.32 (0.97–1.81)	0.079	1.52 (1.12–2.05)	0.007
Eating snacks in front of PC/laptop/tablet, days/week				
<1	Ref.		Ref.	
1–2	1.26 (0.95–1.68)	0.104	1.06 (0.83–1.35)	0.650
3–4	1.69 (1.25–2.28)	0.001	1.33 (0.97–1.83)	0.074
≥5	2.19 (1.71–2.79)	<0.001	1.61 (1.22–2.13)	0.001
Eating meals while watching TV, days/week				
<1	Ref.		Ref.	
1–2	1.28 (1.00–1.64)	0.054	1.03 (0.81–1.30)	0.817
3–4	1.48 (1.11–1.95)	0.007	1.50 (1.13–1.98)	0.005
≥5	1.53 (1.18–1.97)	0.001	1.41 (1.07–1.85)	0.014
Eating in fast-food restaurants				
Rarely	Ref.		Ref.	
1–3 days per month	1.48 (1.19–1.86)	0.001	1.56 (1.26–1.92)	<0.001
Once per week	2.15 (1.6–2.88)	<0.001	2.20 (1.62–2.97)	<0.001
At least twice per week	3.41 (2.15–5.41)	<0.001	8.12 (4.34–15.18)	<0.001
Family affluence scale (FAS) score[a]				

Least (20%) affluent	Ref.		Ref.	
Middle (60%) affluent	0.93 (0.71–1.22)	0.584	0.75 (0.58–0.98)	0.034
Highest (20%) affluent	0.65 (0.47–0.89)	0.007	0.66 (0.48–0.89)	0.006

Abbreviation: Ref.; Reference. [a] Quantiles calculated based on FAS score distribution of FAS score by gender and age group. [†] Diet quality is the outcome variable which is divided into three categories: good, moderate and poor. These categories are dietary score's quantiles calculated as follows; poor: dietary scores from 0 to 9, moderate: dietary scores from 10 to 12, good: dietary scores from 13 to 16. The good diet quality category is the reference category in order to identify behaviors associated with increased odds of not having a good diet quality score.

Table S4. Adjusted odds ratios (OR) and associated 95% confidence intervals (CI) from multivariable ordinal logistic regression model further adjusted for body mass index for the association between diet-related behaviors and other characteristics and diet quality score† among 3386 participants in the 2018 Greek arm of the Health Behaviour of School-Aged Children Study.

**Behaviors/Characteristics**	**Diet Quality score†**	
	**OR (95% CI)**	***p*-Value**
Gender		
Boys	1.36 (1.19–1.55)	<0.001
Girls	Ref.	
Age group		
11-year olds	Ref.	
13-year olds	1.32 (1.10–1.59)	0.003
15-year olds	1.64 (1.35–2.00)	<0.001
Place of birth		
Greece	Ref.	
Other	1.20 (0.82–1.76)	0.355
Body mass index, kg/m^2	0.97 (0.95–0.99)	0.011
Physical activity in the past 7 days, days	0.79 (0.76–0.82)	<0.001
Eating breakfast on weekdays, days/week		
≤1	1.62 (1.39–1.89)	<0.001
2–3	1.25 (1.03–1.52)	0.024
4–5	Ref.	
Eating with family		
Rarely	1.35 (1.13–1.62)	0.001
Almost everyday	Ref.	
Eating snacks while watching TV, days/week		
<1	Ref.	
1–2	1.25 (1.04–1.50)	0.019
3–4	1.52 (1.23–1.87)	<0.001
≥5	1.39 (1.12–1.72)	0.003
Eating snacks in front of PC/laptop/tablet, days/week		
<1	Ref.	
1–2	1.15 (0.96–1.39)	0.131
3–4	1.51 (1.21–1.88)	<0.001
≥5	1.92 (1.59–2.31)	<0.001
Eating meals while watching TV, days/week		
<1	Ref.	
1–2	1.16 (0.97–1.38)	0.096
3–4	1.47 (1.20–1.81)	<0.001
≥5	1.50 (1.24–1.81)	<0.001
Visiting fast-food restaurants		
Rarely	Ref.	
1–3 days per month	1.53 (1.30–1.80)	<0.001

Once per week	2.22 (1.78–2.78)	<0.001
At least twice per week	4.28 (2.89–6.34)	<0.001
Family affluence (FAS) score [a]		
Least affluent 20%	Ref.	
Middle affluent 60%	0.86 (0.72–1.04)	0.123
Highest affluent 20%	0.68 (0.55–0.84)	<0.001

Abbreviation: Ref.; Reference. [a] Quantiles calculated based on gender and age group. † Diet quality is the outcome variable which is divided into three categories: good, moderate and poor. These categories are dietary score's quantiles calculated as follows; poor: dietary scores from 0 to 9, moderate: dietary scores from 10 to 12, good: dietary scores from 13 to 16. The good diet quality category is the reference category in order to identify behaviors associated with increased odds of not having a good diet quality score.
